# Nonlinear relationship between triglyceride glucose-body mass index and the risk of prediabetes: a multi-center retrospective cohort study

**DOI:** 10.3389/fpubh.2026.1916726

**Published:** 2026-09-18

**Authors:** Hui Peng, Zhimao Cai, Yongqiang Zhan, Wei Yang, Xiaomei Zeng, Yuan Dai, Junhong Pan, Rui Jia, Youlian Luo, Hualang Cai, Weifeng Chen, Dan Zhou

**Affiliations:** 1 Department of General Medicine, Shenzhen Second People’s Hospital, The First Affiliated Hospital of Shenzhen University, Shenzhen, Guangdong, China; 2 Shenzhen University Health Science Center, Shenzhen, Guangdong, China; 3 Department of Hepatopancreatobiliary Surgery, Shenzhen Second People’s Hospital, The First Affiliated Hospital of Shenzhen University, Shenzhen, Guangdong, China; 4 Department of Hepatology, National Clinical Research Center for Infectious Diseases, The Third People’s Hospital of Shenzhen, Shenzhen University Health Science Center, Shenzhen, Guangdong, China; 5 Department of Family Medicine, Shenzhen University Medical School; Shenzhen, Guangdong, China

**Keywords:** Chinese, cohort study, nonlinear association, prediabetes, TyG-BMI

## Abstract

**Background:**

The triglyceride-glucose body mass index (TyG-BMI), a novel composite measure integrating lipid-glucose metabolism and obesity, has shown promise in predicting diabetes risk. Nevertheless, its association with the onset of prediabetes remains insufficiently investigated, particularly concerning potential nonlinear relationships.

**Methods:**

Cox proportional hazards models were used to calculate hazard ratios (HRs) and 95% confidence intervals (CIs). Nonlinear relationships were evaluated through the use of smooth curve fitting and threshold effect models. Additionally, interaction tests and subgroup analyses were undertaken to examine the robustness of the associations found.

**Results:**

Following comprehensive adjustment, a 10-unit increment in TyG-BMI correlated with an 11% elevated risk of developing prediabetes (Hazard Ratio [HR] 1.11, 95% Confidence Interval [CI] 1.09–1.13). A significant nonlinear association was observed, with an inflection point identified at a TyG-BMI value of 189.37. Below the threshold, each unit increase in TyG-BMI was linked to a 2% rise in risk (HR 1.02, 95% CI 1.02–1.03), whereas above this point, the risk increase diminished to 1% per unit (HR 1.01, 95% CI 1.01–1.01).

**Conclusion:**

This research identifies a significant, nonlinear association between TyG-BMI and the likelihood of developing prediabetes. Additional research is required to thoroughly examine their relationship and clarify the underlying mechanisms.

## Introduction

Prediabetes is characterized as an intermediary phase of impaired glucose regulation, signifying a heightened risk for the progression to type 2 diabetes. According to estimates by the International Diabetes Federation, approximately 720 million adults globally were affected by prediabetes in 2021, with projections indicating an increase to 1 billion by 2045 (1, 2). The annual risk of transitioning from prediabetes to type 2 diabetes is approximately 10% (3), and this condition is linked with elevated risks of all-cause mortality and cardiovascular events, estimated at approximately 7.36 and 8.75 events per 10,000 person-years, respectively (3). Unlike diagnosed diabetes, prediabetes is often reversible, underscoring the importance of its early detection and management as a critical component of diabetes prevention strategies. The pathogenesis of diabetes involves intricate pathophysiological mechanisms, primarily encompassing insulin resistance and β-cell dysfunction. Among these, insulin resistance is regarded as a pivotal precursor in the disease’s progression (4–6). Consequently, the early identification of individuals at high risk of insulin resistance and prediabetes is essential for the timely implementation of effective preventive interventions (3, 7).

While the hyperinsulinemia-euglycemic clamp technique remains the gold standard for evaluating insulin resistance, its complexity and high cost restrict its widespread clinical use (8–10). This limitation has driven researchers to identify simple and reliable biomarkers that can serve as alternative indicators for assessing insulin resistance in both clinical and public health contexts. The triglyceride-glucose index (TyG), derived from fasting triglyceride and glucose measurements, has become a reliable indicator closely linked to insulin resistance and can forecast prediabetes and diabetes (11–13). Concurrently, obesity, measured by body mass index (BMI), is a recognized contributor to the development of insulin resistance, with dysfunctional adipose tissue being crucial in metabolic disorders (14, 15). In light of the synergistic relationship between metabolic disorders and obesity, researchers have recently introduced the “TyG-BMI index,” which is computed as the product of the TyG index and BMI. A growing body of evidence from cross-sectional and retrospective cohort studies demonstrates that TyG-BMI exhibits superior predictive performance for future type 2 diabetes risk compared to either of its individual components (16, 17). This composite index integrates the metabolic dysregulation reflected by the TyG index with the anthropometric burden measured by BMI, thereby providing a more comprehensive assessment of an individual’s metabolic status (18, 19).

Although emerging evidence has linked the TyG-BMI index to the onset, progression, and regression of prediabetes, the precise dose–response relationship remains insufficiently characterized (3, 20). Understanding this association is critical for addressing prediabetes. From a biological standpoint, we postulated that the relationship between TyG-BMI and the risk of prediabetes might demonstrate a nonlinear pattern, potentially exhibiting a saturation effect beyond a specific threshold. Consequently, this study hypothesizes that there is an association between TyG-BMI and the risk of prediabetes. We employ smooth curve fitting and threshold effect to assess nonlinearity. If present, we apply segmented regression model to determine the inflection point. This analysis is anticipated to provide a more profound understanding of the dynamic changes linked to prediabetes.

## Methods

### Study design and data source

This study utilizes a secondary analysis of a pre-existing dataset as its research design. The initial study was a retrospective cohort analysis, and the data used for this research are available to the public through the Dryad digital repository (DOI:10.5061/dryad.ft8750v) (21). The original cohort was established by the Rich Healthcare Group in China, which maintains a computerized database of health checkup records from 2010 to 2016. Data were collected from 32 sites across 11 cities, including Shanghai, Beijing, Nanjing, Shenzhen, and Guangzhou. At each visit, participants completed standardized questionnaires and underwent anthropometric measurements and laboratory testing on fasting blood samples, with rigorous quality control procedures. The original group included 685,277 individuals from China, each aged 20 or above, with a minimum of two documented medical appointments between 2010 and 2016. The follow-up period began after the initial health check in 2010 and ended either when diabetes was diagnosed or on December 31, 2016. Following Chen’s research framework, specific participants were omitted from this retrospective cohort study due to the following initial criteria: (1) a prior diagnosis of diabetes, (2) a follow-up period of less than 2 years, (3) an unknown diabetes status during the follow-up period, (4) unavailable weight and height measurements, (5) missing gender information, (6) unavailable fasting blood glucose (FPG) values, and (7) had extreme levels of body mass index (BMI) values (BMI > 55 kg/m^2^ or BMI < 15 kg/m^2^). This comprehensive data collection ultimately constituted an initial dataset comprising 211,833 individuals.

In the present analysis, we leveraged this dataset to examine the association between the triglyceride-glucose body mass index (TyG-BMI) and the risk of developing prediabetes. For the secondary analysis presented in this article, we further refined the original sample by applying exclusion criteria consistent with those used in previous studies that employed the same Rich Healthcare Group database for prediabetes (22). Specifically, 32,656 individuals were excluded based on the following criteria: 26,247 individuals exhibited fasting plasma glucose (FPG) levels of ≥5.6 mmol/L at baseline; 4,269 individuals lacked available triglyceride (TG) data; 856 individuals presented with extreme TG values (TG = 0 mmol/L); in line with the standard practice adopted in the aforementioned study (22), we also excluded 1,248 individuals who were diagnosed with diabetes during follow-up and 36 individuals who had FPG levels >6.9 mmol/L during follow-up. Consequently, the final sample comprised 179,177 participants, as illustrated in Figure 1.

**Figure 1:**
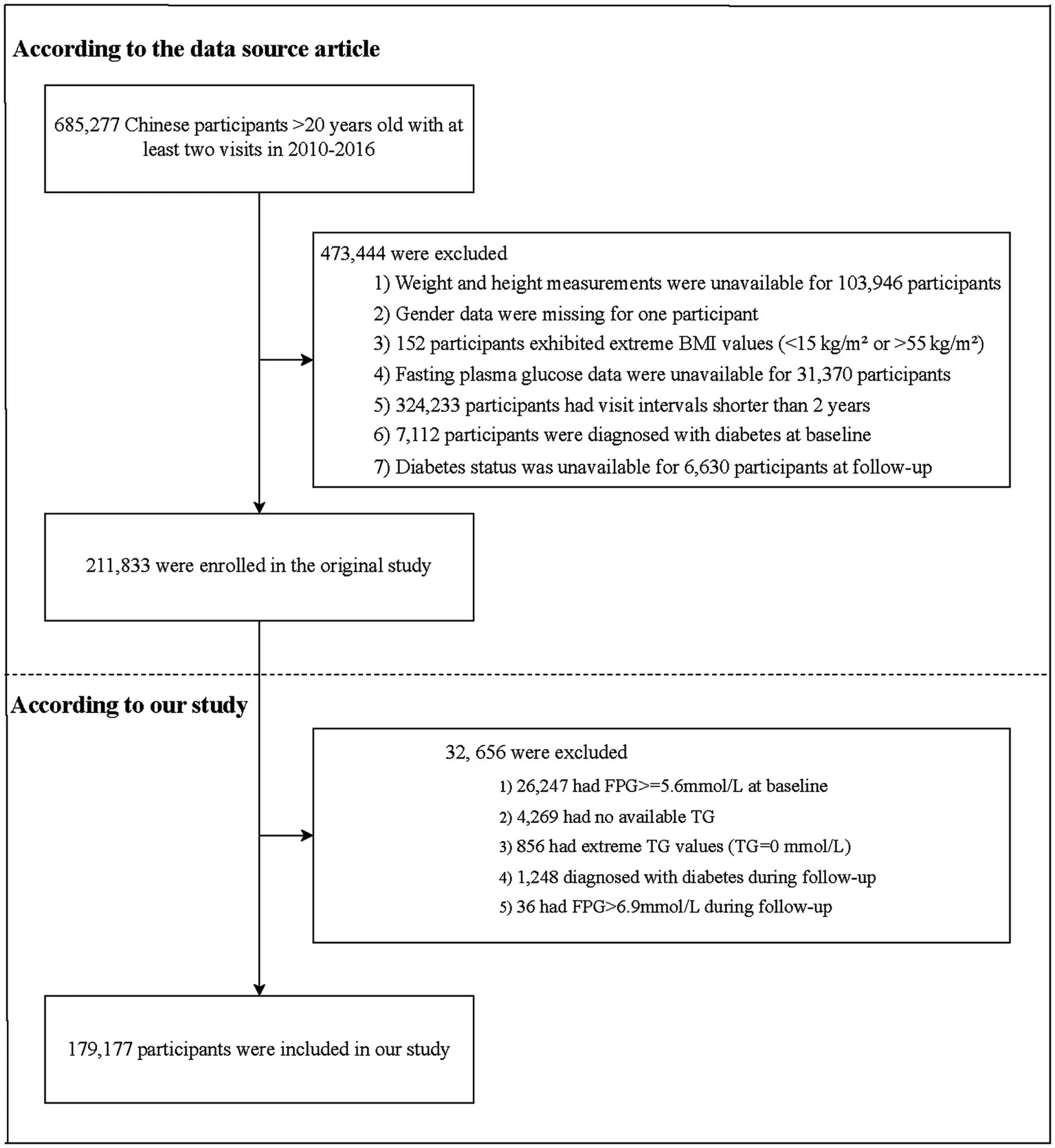
Flow diagram of subjects included in the cohort study. FPG, fasting blood glucose; TG, triglycerides.

Additionally, comprehensive data on various indicators were collected in previous studies, including demographic information (age, gender), lifestyle factors (smoking and alcohol consumption status), chronic disease history (family history of diabetes), and laboratory test results [diastolic blood pressure (DBP),systolic blood pressure (SBP), body mass index (BMI),triglycerides (TG),high-density lipoprotein cholesterol (HDL-C), low-density lipoprotein cholesterol(LDL-C), blood urea nitrogen (BUN),alanine aminotransferase (ALT), aspartate aminotransferase (AST), and serum creatinine (SCR),fasting blood glucose (FPG)].

The classification of smoking status was as follows: 1 for current smokers, 2 for former smokers, and 3 for individuals who have never smoked. Similarly, drinking status was categorized as 1 for current drinkers, 2 for former drinkers, and 3 for those who have never consumed alcohol. The design of the retrospective cohort study was meticulously documented in the initial research (21).

This study was approved by the Rich Healthcare Group Review Board. Since this research involved only the analysis of anonymized, pre-existing data sourced from the Dryad database, additional ethical review was deemed unnecessary. The initial data collection methods complied with the guidelines of the Declaration of Helsinki and were sanctioned by the ethics committee of the authors’ affiliated institution (21).

### Exposure measures

TyG-BMI is calculated by multiplying BMI with the TyG index, where the TyG index is the natural logarithm of [FPG (mg/dL) times TG (mg/dL) divided by 2], and BMI is determined by dividing weight by height squared (kg/m^2^) (19).

### Outcome measures

Prediabetes is defined by fasting glucose levels ranging from 5.6 to 6.9 mmoL/L (22).

### Statistical analysis

Statistical analyses were performed using EmpowerStats software^1^ (X&Y Solutions Inc., Boston, Massachusetts, United States). A *p*-value of less than 0.05 was used to determine statistical significance.

The TyG-BMI levels were stratified into quartiles for analytical purposes: the first quartile (96.66–164.74), the second quartile (164.74–187.90), the third quartile (187.90–214.86), and the fourth quartile (214.86–477.03). Variables that follow a normal distribution were expressed as mean ± standard deviation, while those with non-normal distributions were shown as median (interquartile range). Percentages were used to express categorical variables. Suitable statistical tests were applied to evaluate baseline characteristics across the four quartiles, depending on the variables’ distribution and nature. Specifically, one-way ANOVA was conducted on continuous variables that were normally distributed, while the Kruskal-Wallis test was applied to data lacking normal distribution. For categorical variables, differences in proportions between groups were evaluated using the Chi-square test.

The survival rates and cumulative event rates across various TyG-BMI groups were analyzed utilizing the Kaplan–Meier method. Univariate Cox regression analysis was used to evaluate the effect of TyG-BMI levels on the risk of prediabetes. To gain deeper insights into the association and verify the consistency of the findings, the following models were used in a multivariate Cox regression analysis: Model 1 (unadjusted), Model 2 (adjusted for gender, age, systolic blood pressure [SBP], and diastolic blood pressure [DBP]), and Model 3 (adjusted for gender, age, SBP, DBP, alcohol consumption, smoking status, family history of diabetes, high-density lipoprotein cholesterol [HDL-C],aspartate aminotransferase [AST], alanine aminotransferase [ALT], serum creatinine [Scr],and blood urea nitrogen [BUN]).

To examine the potential non-linear link between TyG-BMI and prediabetes onset, researchers utilized a Cox proportional hazards regression model with smooth curve fitting. Inflection points were identified using a recursive algorithm, followed by the development of two segmented Cox proportional hazards regression models to independently assess data on both sides of these points. Researchers used the log-likelihood ratio test to pinpoint the most suitable model for the connection between TyG-BMI and prediabetes onset.

To examine potential effect modifications, we conducted interaction and stratified analyses by clinically relevant subgroups, including age (<60 vs. ≥60 years), sex, family history of diabetes, smoking status and drinking status. As a sensitivity check for our primary results, we handled missing data using a two-tier strategy: complete-case analysis was employed when the overall missing rate fell below 5%; otherwise, we performed multiple imputation using the chain-equation method with five replications, re-ran the main analyses on the imputed datasets, and pooled the results according to Rubin’s rules (23, 24).

## Results

### The characteristics of participants

In an analysis of 179,177 participants categorized by TyG-BMI quartiles, baseline characteristics exhibited significant trends (all *p*-values <0.001) as detailed in Table 1. As TyG-BMI values increased from the first quartile (96.66–164.74) to the fourth quartile (214.86–477.03), participants were characterized by older age, elevated blood pressure, more adverse lipid profiles, increased liver enzyme levels, and a higher prevalence of males, current smokers, and alcohol consumers. Notably, the prevalence of prediabetes showed a pronounced graded increase, escalating from 5.13% in the first quartile to 18.92% in the fourth quartile, indicating an approximate four-fold rise.

**Table 1:** The characteristics of participants.

TyG-BMI quartile	Q1 (96.66–164.74)	Q2 (164.74–187.90)	Q3 (187.90–214.86)	Q4 (214.86–477.03)	*p*-value
Participants	44,794	44,794	44,794	44,795	
Age (years)	36.24 ± 9.68	40.28 ± 11.59	43.49 ± 12.63	44.48 ± 12.68	<0.001
SBP (mmHg)	110.38 ± 13.23	114.93 ± 14.49	120.17 ± 15.15	125.91 ± 15.92	<0.001
DBP (mmHg)	69.11 ± 9.04	71.39 ± 9.58	74.75 ± 10.12	78.87 ± 10.95	<0.001
ALT (U/L)	13.00 (10.20, 17.00)	15.10 (11.70, 21.00)	19.50 (14.30, 27.80)	27.50 (19.00, 41.00)	<0.001
Scr (mmol/L)	63.28 ± 13.33	67.54 ± 15.77	72.18 ± 15.20	75.59 ± 15.74	<0.001
LDL-C (mmol/L)	2.50 ± 0.58	2.68 ± 0.63	2.84 ± 0.67	2.92 ± 0.70	<0.001
TC (mmol/L)	4.35 ± 0.78	4.56 ± 0.83	4.77 ± 0.87	5.00 ± 0.90	<0.001
BMI (kg/m^2^)	19.32 ± 1.32	21.75 ± 1.17	23.85 ± 1.29	27.07 ± 2.33	<0.001
FPG (mmol/L)	4.63 ± 0.48	4.74 ± 0.47	4.82 ± 0.46	4.90 ± 0.45	<0.001
TG (mmol/L)	0.66 (0.51, 0.84)	0.89 (0.70, 1.14)	1.20 (0.91, 1.58)	1.83 (1.35, 2.52)	<0.001
Gender					<0.001
Male	12,551 (28.02%)	19,868 (44.35%)	28,203 (62.96%)	34,504 (77.03%)	
Female	32,243 (71.98%)	24,926 (55.65%)	16,591 (37.04%)	10,291 (22.97%)	
AST					<0.001
Low	8,715 (47.27%)	7,392 (39.51%)	5,336 (28.15%)	3,216 (17.13%)	
Middle	6,377 (34.59%)	6,609 (35.32%)	6,829 (36.03%)	5,385 (28.68%)	
High	3,345 (18.14%)	4,709 (25.17%)	6,791 (35.83%)	10,173 (54.19%)	
BUN					<0.001
Low	16,599 (41.03%)	14,785 (36.21%)	12,204 (29.79%)	10,690 (26.13%)	
Middle	12,900 (31.88%)	13,300 (32.57%)	13,716 (33.48%)	13,824 (33.79%)	
High	10,960 (27.09%)	12,750 (31.22%)	15,048 (36.73%)	16,396 (40.08%)	
HDL-C					<0.001
Low	3,662 (15.76%)	6,059 (24.24%)	9,531 (36.37%)	13,123 (50.25%)	
Middle	7,475 (32.16%)	8,948 (35.80%)	9,445 (36.04%)	8,158 (31.24%)	
High	12,106 (52.08%)	9,984 (39.95%)	7,233 (27.60%)	4,837 (18.52%)	
Smoking status					<0.001
Current smoker	1,039 (9.23%)	1740 (14.66%)	2,632 (20.63%)	3,900 (28.74%)	
Ever smoker	237 (2.10%)	412 (3.47%)	629 (4.93%)	779 (5.74%)	
Never smoker	9,984 (88.67%)	9,719 (81.87%)	9,496 (74.44%)	8,892 (65.52%)	
Drinking status					<0.001
Current drinker	85 (0.75%)	157 (1.32%)	281 (2.20%)	450 (3.32%)	
Ever drinker	879 (7.81%)	1,481 (12.48%)	2,129 (16.69%)	2,729 (20.11%)	
Never drinker	10,296 (91.44%)	10,233 (86.20%)	10,347 (81.11%)	10,392 (76.58%)	
Family history of diabetes					0.001
No	43,996 (98.22%)	43,830 (97.85%)	43,910 (98.03%)	43,904 (98.01%)	
Yes	798 (1.78%)	964 (2.15%)	884 (1.97%)	891 (1.99%)	
Prediabetes					<0.001
No	42,498 (94.87%)	41,062 (91.67%)	39,051 (87.18%)	36,318 (81.08%)	
Yes	2,296 (5.13%)	3,732 (8.33%)	5,743 (12.82%)	8,477 (18.92%)	

Values are *n* (%) or mean ± standard deviation or medians (quartile interval). TyG-BMI, triglyceride glucose-body mass index; SBP, systolic blood pressure; DBP, diastolic blood pressure; BMI, body mass index; TC, total cholesterol; TG, triglycerides; HDL-C, high-density lipoprotein cholesterol; LDL-C, low-density lipoprotein cholesterol; ALT, alanine aminotransferase; AST, aspartate aminotransferase; Scr, serum creatinine; BUN, blood urea nitrogen; FPG, fasting plasma glucose. Among the 179,177 participants, SBP had 16 missing values, DBP had 17 missing values, TC had 1 missing values, HDL-C had 78,616 (43.88%) missing values, LDL-C had 78,113 (43.60%) missing values, ALT had 1,410 (0.79%) missing values, AST had 104,300 (58.21%) missing values, BUN had 16,005 (8.93%) missing values, Scr had 8,193 (4.57%) missing values, smoking status had 129,718 (72.4%) missing values, and drinking status had 129,718 (72.4%) missing values.

Figure 2 illustrates the Kaplan–Meier curves, stratified by TyG-BMI categories, depicting the risk of prediabetes onset. Significant differences were observed among the four TyG-BMI groups regarding prediabetes onset risk (*p* < 0.001), with the cumulative risk of prediabetes onset rising in accordance with increasing TyG-BMI levels.

**Figure 2:**
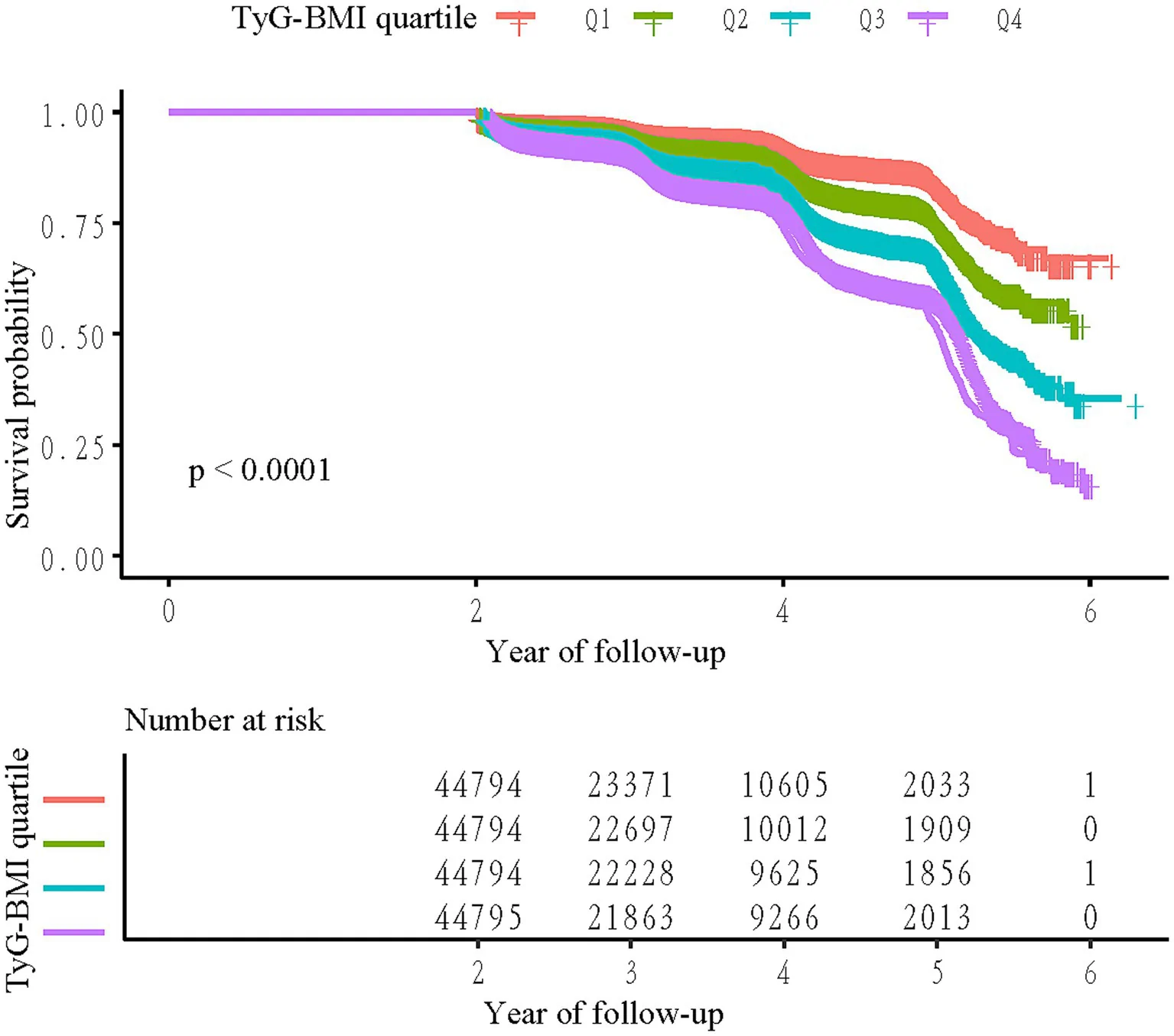
Kaplan–Meier event-free survival curve. Kaplan–Meier analysis of incident prediabetes-free survival based on TyG-BMI groups (log-rank, *p* < 0.0001).

### Relationship between TyG-BMI and incident prediabetes in different models

In multivariable Cox regression analyses, a significant and dose-dependent association was identified between TyG-BMI and the risk of developing prediabetes, as presented in Table 2. In the fully adjusted model (Model 3), a 10-unit increase in TyG-BMI corresponded to an 11% elevated risk (HR 1.11, 95% CI 1.09–1.13, *p* < 0.0001). Analysis by quartiles demonstrated a pronounced graded relationship, with a highly significant trend test (*p* for trend <0.0001). Participants in the highest quartile (Q4) exhibited a risk more than three times greater than those in the lowest quartile (Q1) (HR 3.21, 95% CI 2.52–4.09, *p* < 0.0001). This positive association remained robust after sequential adjustments for demographic, clinical, and lifestyle confounders.

**Table 2:** Relationship between TyG-BMI and incident prediabetes in different models.

Exposure	Model 1 (HR, 95% CI, *p*)	Model 2 (HR, 95% CI, *p*)	Model 3 (HR, 95% CI, *p*)
TyG-BMI (per 10 units)	1.13 (1.13, 1.14) < 0.0001	1.09 (1.09, 1.10) < 0.0001	1.11 (1.09, 1.13) < 0.0001
TyG-BMI (quartile)			
Q1	Ref.	Ref.	Ref.
Q2	1.68 (1.60, 1.77) < 0.0001	1.42 (1.34, 1.49) < 0.0001	1.72 (1.35, 2.19) < 0.0001
Q3	2.65 (2.52, 2.78) < 0.0001	1.90 (1.81, 2.00) < 0.0001	2.46 (1.94, 3.12) < 0.0001
Q4	3.95 (3.77, 4.14) < 0.0001	2.54 (2.42, 2.68) < 0.0001	3.21 (2.52, 4.09) < 0.0001
*p* for trend	<0.0001	<0.0001	<0.0001

Model 1: unadjusted; Sample size (*n* = 179,177).

Model 2: Model 1 + gender, age SBP and DBP; Sample size (*n* = 179,160).

Model 3: Model 2 + family history of diabetes, drinking status, smoking status, HDL-C, Scr, BUN, ALT and AST. Sample size (*n* = 10,545).

HR, hazard ratios; CI, confidence interval; Ref, reference.

### The relationship between TyG-BMI levels and incident prediabetes

Upon fitting smoothing splines, the relationship between TyG-BMI and the risk of prediabetes was found to be non-linear (Figure 3). A threshold effect analysis revealed a significant nonlinear relationship, with an inflection point at a TyG-BMI of 189.37. Below this threshold, each unit increase in TyG-BMI was associated with a 2% increase in the hazard of prediabetes (HR 1.02, 95% CI 1.02–1.03, *p* < 0.0001). Beyond this inflection point, the risk continued to escalate, albeit at a marginally reduced rate of 1% per unit increase (HR 1.01, 95% CI 1.01–1.01, *p* < 0.0001). The log-likelihood ratio test substantiated that this two-piecewise linear model offered a significantly superior fit compared to a simple linear model (*p* < 0.001), thereby reinforcing the existence of a nonlinear relationship (Table 3).

**Figure 3:**
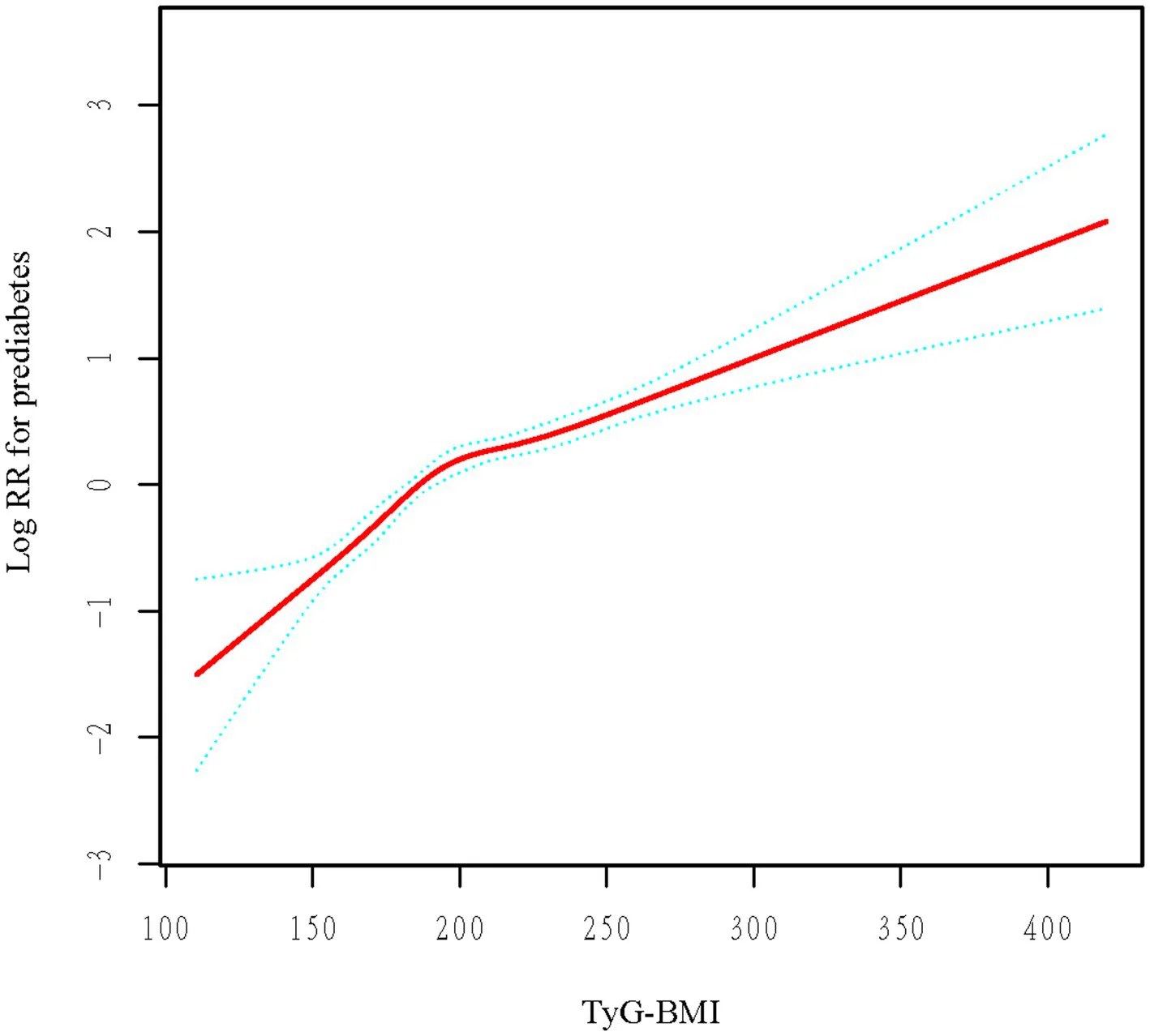
The relationship between TyG-BMI and incident prediabetes. A linear relationship between them was detected after adjusting for age, gender, SBP, DBP, family history of diabetes, drinking status, smoking status, HDL-C, Scr, BUN, ALT and AST. The red curve on the graph represents the smoothed fit between the variables, while the blue lines indicate the 95% confidence intervals surrounding the fit.

**Table 3:** Threshold effect analysis between TyG-BMI and incident prediabetes.

Threshold effect analysis	Outcome HR (95% CI) *p*-value
Inflection point of TyG-BMI (K)	189.37
<K slope	1.02 (1.02, 1.03) < 0.0001
>K slope	1.01 (1.01, 1.01) < 0.0001
Log-likelihood ratio test	<0.001

The above model was adjusted for gender, age, SBP, DBP, family history of diabetes, drinking status, smoking status, HDL-C, Scr, BUN, ALT and AST.

### Subgroup analyses

An extensive subgroup analysis was conducted to assess the consistency of the association between TyG-BMI and prediabetes risk across diverse populations (Table 4). The positive association was consistently observed across all predefined subgroups, including those categorized by age, gender, smoking status, alcohol consumption, and family history of diabetes (all *p* < 0.05, with the exception of current drinkers: HR 1.09, 95% CI 1.00–1.20, *p* = 0.055). Notably, no significant interaction was identified for any stratification variable (all *p* for interaction >0.05), indicating that the strength of the association between TyG-BMI and prediabetes risk remains statistically consistent.

**Table 4:** Subgroup analysis.

Characteristic	HR (95%CI)	*p*-value	*p* for interaction
Age			0.6509
<60	1.12 (1.10, 1.14)	<0.0001	
> = 60	1.11 (1.05, 1.16)	<0.0001	
Gender			0.4208
Male	1.11 (1.09, 1.13)	<0.0001	
Female	1.12 (1.09, 1.16)	<0.0001	
Smoking status			0.9013
Current smoker	1.11 (1.07, 1.14)	<0.0001	
Ever smoker	1.12 (1.06, 1.20)	0.0002	
Never smoker	1.11 (1.09, 1.13)	<0.0001	
Drinking status			0.9265
Current drinker	1.09 (1.00, 1.20)	0.0554	
Ever drinker	1.11 (1.07, 1.15)	<0.0001	
Never drinker	1.11 (1.09, 1.14)	<0.0001	
Family history of diabetes			0.3196
No	1.11 (1.09, 1.14)	<0.0001	
Yes	1.08 (1.03, 1.14)	0.0033	

For each 10-unit increase in the TyG-BMI. The above model was adjusted for gender, age, SBP, DBP, family history of diabetes, drinking status, smoking status, HDL-C, Scr, BUN, ALT and AST; and the model was not adjusted for the stratification variable in each case.

### Sensitivity analysis

To further validate the robustness of our primary findings, we conducted a series of sensitivity analyses. First, to exclude the possibility that the observed association was influenced by baseline glycemia, we included continuous baseline FPG as a covariate in the main model. The nonlinear association between TyG-BMI and the risk of prediabetes remained robust (Supplementary Figure S1 and Table S1). In addition, we performed ROC curve analyses for TyG, BMI, and TyG-BMI (Supplementary Figure S2). To address the concern of missing data, we repeated the main analyses (Model 3) after multiple imputation. After imputation, the non-linear association persisted, with effect sizes on both sides of the breakpoint remaining consistent (Supplementary Table S2).

## Discussion

In this large-scale retrospective cohort study, we identified a robust and independent positive association between the TyG-BMI index and the risk of incident prediabetes. The principal findings are as follows: Firstly, a fully adjusted Cox proportional hazards model indicated a significant positive association, with each 10-unit increase in TyG-BMI correlating with an 11% heightened risk of developing prediabetes. Secondly, a clear dose–response link was observed, as individuals in the highest TyG-BMI quartile demonstrated a risk more than three times greater than those in the lowest quartile. Thirdly, and most notably, this association was nonlinear. A threshold effect analysis revealed a significant inflection point at a TyG-BMI of 189.37. Below this threshold, each unit increase in TyG-BMI was associated with a 2% increase in risk, whereas above this threshold, the risk increase per unit was reduced to 1%. Lastly, the consistency of this association was validated through comprehensive subgroup analyses, which showed that the positive relationship remained statistically significant and stable across various strata defined by age, gender, smoking status, and other key covariates.

In the field of prediabetes research, biomarkers associated with lipid and glucose metabolism have increasingly garnered scholarly attention. Notably, TyG index has emerged as a robust indicator for evaluating insulin resistance, demonstrating a strong correlation with prediabetes. Empirical studies have suggested that integrating the TyG index with obesity metrics, such as BMI, enhances predictive accuracy, particularly in the detection of new prediabetes and diabetes cases (25–27). Further research indicates that the predictive capability of the TyG-BMI composite measure for insulin resistance surpasses that of isolated glucose or lipid indicators (19, 28). This study, utilizing a large-scale retrospective cohort analysis, corroborates the significant link between TyG-BMI and the risk of prediabetes onset, aligning with recent trends in this research domain. Importantly, the previous assumption of a linear relationship may have obscured the true dose–response association between TyG-BMI and the risk of diabetes. Utilizing smooth curve fitting and threshold effect models, this study—to our knowledge, for the first time—elucidated a significant nonlinear relationship between TyG-BMI and the risk of prediabetes, identifying the inflection point value as 189.37.

Drawing on extensive cohort data, the research confirmed a substantial association between TyG-BMI and prediabetes risk, underpinned by a well-established pathophysiological basis. As a composite indicator, TyG-BMI encapsulates two fundamental aspects of diabetes progression: insulin resistance, as represented by the TyG index, and obesity, as indicated by BMI. Previous studies have demonstrated that excessive adipose tissue accumulation promotes the release of pro-inflammatory factors, which disrupt the insulin signaling pathway and exacerbate insulin resistance (14, 29–31). Additionally, adipose tissue functions as an active endocrine organ, capable of secreting inflammatory factors such as TNF-α and IL-6 in the context of obesity, thereby inducing chronic low-grade inflammation and further intensifying systemic insulin resistance (32, 33). As metabolic burden intensifies, changes in the slope around the inflection point were observed, and the incremental risk of prediabetes associated with further increases in TyG-BMI attenuated beyond this point. This finding provides preliminary evidence for the association between metabolic alterations and prediabetes risk, although its direct clinical utility warrants further evaluation in future prospective or interventional studies.

This study offers several notable methodological strengths. Firstly, utilizing a large-scale retrospective cohort design, we included over 170,000 participants and conducted long-term follow-up, thereby providing robust evidence for examining the temporal link between TyG-BMI and prediabetes, which significantly enhances the reliability of causal inference. Secondly, by collecting comprehensive baseline data encompassing demographic characteristics, biochemical indicators, lifestyle factors, and family history, and employing a multivariate Cox proportional hazards model for systematic adjustment, we effectively minimized potential confounding biases. Particularly noteworthy is our application of advanced statistical techniques, such as smooth curve fitting and piecewise regression, which, for the first time, elucidated the non-linear connection between TyG-BMI and prediabetes risk, identifying the critical turning point of 189.37 and thereby deepening our understanding of this association. Furthermore, extensive subgroup analyses and interaction tests demonstrated that this association remained consistent across various subgroups, including age, gender, and smoking status, further corroborating the strength of our results.

While this study makes significant contributions, several limitations must be acknowledged. First, the observational nature of the study design constrains the ability to establish definitive causal relationships. Second, the data used in this study were collected nearly a decade ago, during which substantial changes may have occurred in population health status, lifestyle factors, and healthcare practices. Consequently, caution should be exercised when directly extrapolating the fundamental biological relationships identified herein to contemporary populations. Future prospective studies utilizing more recent multi-center cohort data are warranted to validate these findings and update risk estimates within the context of current public health and clinical practice. Third, it should be noted that TyG-BMI inherently incorporates baseline FPG, and the prediabetes outcome is also defined by FPG during follow-up. To address this, we included continuous baseline FPG as a covariate in the fully adjusted model, and the nonlinear association between TyG-BMI and incident prediabetes remained consistent. Fourth, the TyG-BMI was assessed solely at baseline, which precludes consideration of its dynamic changes over time. Fifth, the original cohort on which our secondary analysis was based applied stringent exclusion criteria (including missing data, insufficient follow-up, baseline diabetes, and extreme BMI values), resulting in the exclusion of 473,442 participants. Although we further restricted the sample according to our own research hypotheses, we acknowledge that these exclusions may have introduced selection bias. Future studies are warranted to validate our findings in broader populations. Furthermore, the original dataset lacked comprehensive data on dietary intake and physical activity levels—key upstream determinants of TyG-BMI involved in lipid-glucose metabolism and cardiometabolic health (34, 35)—thereby precluding adjustment for these factors. As a result, residual confounding from these unmeasured factors cannot be entirely dismissed. Finally, we excluded individuals who developed new-onset diabetes (*n* = 1,248) or had FPG > 6.9 mmol/L (*n* = 36) during follow-up. Although this approach followed the standard practice of comparable studies to ensure endpoint purity (22), it may limit the generalizability of our findings, and caution should be exercised when extrapolating the results. Future studies should employ multi-center, longitudinal designs with comprehensive assessments of lifestyle and medication use to validate these findings.

## Conclusion

In conclusion, the findings of this research indicate a significant nonlinear association between TyG-BMI and prediabetes risk. These findings offer preliminary evidence that may inform future risk assessment for prediabetes, though additional studies are warranted to thoroughly characterize this relationship and clarify its mechanistic basis.

## Data Availability

Publicly available datasets were analyzed in this study. This data can be found here: https://datadryad.org/stash/dataset/doi:10.5061%2Fdryad.ft8750v.

## Footnotes

1
http://www.empowerstats.com/cn/
