# Supplementary material for: Nonlinear relationship between triglyceride glucose-body mass index and the risk of prediabetes: a multi-center retrospective cohort study
Source: Front Public Health. 2026 Sep 18;14:1916726. doi: 10.3389/fpubh.2026.1916726 (PMC13630975; doi:10.3389/fpubh.2026.1916726)
Supplement: Supplementary file 2 [file Table_1.DOCX]

**Supplemental Materials**

**Supplementary Figures**

Figure S1. The relationship between TyG-BMI and incident prediabetes	2

Figure S2. Receiver operating characteristic (ROC) curve for BMI, TyG and TyG-BMI in incident prediabetes	3

**Supplementary Tables**

Table S1. Threshold effect analysis between TyG-BMI and incident prediabetes	2

Table S2. Pooled threshold effect analysis between TyG-BMI and incident prediabetes following multiple imputation	3

**Figure S1.**

**Figure S1.** The relationship between TyG-BMI and incident prediabetes. A linear relationship between them was detected after adjusting for gender, age, SBP, DBP, family history of diabetes, drinking status, smoking status, HDL-C, Scr, BUN, ALT, AST and FPG. The red curve on the graph represents the smoothed fit between the variables, while the blue lines indicate the 95% confidence intervals surrounding the fit.

**Table S1. Threshold effect analysis between TyG-BMI and incident prediabetes**

| Threshold effect analysis | Outcome |
| --- | --- |
|  | HR (95%CI) p-value |
| Inflection point of TyG-BMI (K) | 182.45 |
| <K slope | 1.01 (1.01, 1.02) <0.0001 |
| >K slope | 1.00 (1.00, 1.01) <0.0001 |
| Log-likelihood ratio test | 0.014 |

**Note:** The above model was adjusted for gender, age, SBP, DBP, family history of diabetes, drinking status, smoking status, HDL-C, Scr, BUN, ALT, AST and FPG.

**Figure S2.**

**Figure S2.** Receiver operating characteristic (ROC) curve for BMI, TyG and TyG-BMI in incident prediabetes. The area under the curve (AUC) are presented.

**Table S2.** **Pooled threshold effect analysis between TyG-BMI and incident prediabetes following multiple imputation**

| Threshold effect analysis | Outcome |
| --- | --- |
|  | HR (95%CI) p-value |
| Inflection point of TyG-BMI (K) | 189.37 |
| <K slope | 1.02 (1.01, 1.02) <0.0001 |
| >K slope | 1.01 (1.01, 1.01) <0.0001 |
| Log-likelihood ratio test | <0.001 |

**Note:** Results were pooled from 5 imputed datasets according to Rubin's rules. The effect sizes on both sides of the breakpoint are consistent after imputation. The above model was adjusted for gender, age, SBP, DBP, family history of diabetes, drinking status, smoking status, HDL-C, Scr, BUN, ALT and AST. Results were pooled from 5 imputed datasets.
